# Multiple Extensive Odontogenic Keratocysts in a Child With Gorlin-Goltz Syndrome: A Case Report

**DOI:** 10.7759/cureus.116403

**Published:** 2026-09-17

**Authors:** Jeremi Matysek, Dominika Lech, Robert Maksymowicz, Cyprian Straczek, Krzysztof Dowgierd

**Affiliations:** 1 Department of Clinical Pediatrics, Head and Neck Surgery Clinic for Children and Young Adults, University of Warmia and Mazury in Olsztyn, Olsztyn, POL; 2 Department of Maxillofacial, Reconstructive and Plastic Surgery, Head and Neck Surgery Clinic for Children and Young Adults, Regional Specialized Children’s Hospital in Olsztyn, Olsztyn, POL

**Keywords:** case report, gorlin-goltz syndrome, multiple odontogenic keratocysts, odontogenic keratocyst, pediatric maxillofacial surgery, ptch1

## Abstract

Gorlin-Goltz syndrome (GGS) is a rare autosomal dominant multisystem disorder characterized by a broad spectrum of developmental abnormalities and tumor predisposition. Multiple odontogenic keratocysts (OKCs) are one of its major diagnostic manifestations and may precede the development of other characteristic features. Early recognition of syndromic OKCs is particularly important in children, in whom other clinical manifestations may not yet be apparent.

We present the case of a 13-year-old boy referred for evaluation of progressive facial asymmetry caused by extensive maxillary lesions. Maxillofacial computed tomography revealed four large, well-defined osteolytic lesions involving both maxillae and the mandible, with associated impacted teeth. The largest lesions occupied nearly the entire maxillary sinuses and caused marked cortical thinning, expansion, and displacement of adjacent structures. Magnetic resonance imaging confirmed the presence of four lesions consistent with OKCs. Additional imaging demonstrated lamellar calcification of the falx cerebri and characteristic rib abnormalities. Based on the presence of two major diagnostic criteria, OKCs diagnosed before 20 years of age and lamellar calcification of the falx cerebri, and one minor criterion, rib abnormalities, a clinical diagnosis of GGS was established according to current diagnostic guidelines. Genetic evaluation was initiated and was ongoing at the time of manuscript preparation. All four lesions were surgically enucleated during a single procedure, together with the associated impacted or ectopically positioned teeth. Histopathological examination confirmed the diagnosis of OKCs in all excised lesions. The postoperative course was uncomplicated, and the patient was referred for continued multidisciplinary follow-up because of the risk of recurrence and development of additional lesions.

This case highlights the importance of considering GGS in children presenting with multiple OKCs, even in the absence of characteristic cutaneous manifestations. Comprehensive radiological assessment can facilitate recognition of additional diagnostic features and enable early clinical diagnosis, appropriate treatment, and long-term surveillance.

## Introduction

Gorlin-Goltz syndrome (GGS) is an uncommon inherited multisystem disorder, with population estimates suggesting a prevalence between approximately 1 in 31,000 and 1 in 256,000 individuals [1,2]. The syndrome is most commonly associated with pathogenic germline variants affecting PTCH1, a tumor suppressor gene involved in the regulation of the Hedgehog signaling pathway [3,4].

Odontogenic keratocysts (OKCs) are benign jaw lesions that may remain clinically silent in their early stages. However, their progressive growth can result in displacement of adjacent teeth, cortical bone destruction, and other local complications [5]. OKCs occur in approximately 44-92% of patients with basal cell nevus syndrome and may develop from around eight years of age, particularly during the transition from primary to permanent dentition [6,7].

Multiple OKCs are a characteristic manifestation of GGS and may be the first clinically apparent feature of the syndrome, particularly in children in whom other manifestations may not yet be evident. Syndromic OKCs may develop at multiple sites of the jaws, either synchronously or metachronously. Their recognition should therefore prompt comprehensive clinical and radiological evaluation for additional features of GGS [5,8].

In this report, we present a 13-year-old boy with four extensive OKCs involving both maxillae and the mandible. The lesions were associated with intracranial calcifications and characteristic rib abnormalities, supporting a clinical diagnosis of GGS despite the absence of significant symptoms and characteristic cutaneous manifestations. The extensive bilateral involvement of the maxillary sinuses, associated impacted and ectopically positioned teeth, and simultaneous surgical management of all four lesions illustrate the diagnostic and therapeutic challenges of extensive multicentric OKCs in a pediatric patient.

## Case presentation

A 13-year-old boy was electively admitted to the Department of Maxillofacial Surgery because of an extensive lesion of the left maxilla. According to his mother, the patient had not experienced significant pain or other concerning symptoms. Approximately three months before admission, she noticed progressive facial asymmetry. The patient was initially referred by his primary care physician to an otolaryngologist, who subsequently ordered maxillofacial computed tomography (CT) and referred the patient to a specialized maxillofacial surgery center. An earlier CT examination had also been performed at another institution; however, it did not encompass the entire maxillofacial region.

The patient's medical history was otherwise unremarkable. He had no known chronic diseases, was not receiving any regular medications, and had no known drug or food allergies. His vaccinations were reported to be up to date according to the national immunization schedule. There had been no recent infection or antibiotic therapy during the six weeks preceding admission. The patient was born from the fourth pregnancy, which was a twin pregnancy, by vaginal delivery at 38 weeks of gestation. There was no reported family history of similar lesions or features suggestive of GGS.

On clinical examination, facial asymmetry associated with maxillary enlargement was observed. Because of the clinical suspicion of GGS, a dermatological consultation was performed; however, no characteristic cutaneous abnormalities were identified. The patient was subsequently referred for further multidisciplinary evaluation.

A non-contrast CT examination of the maxillofacial region demonstrated four well-defined, unilocular osteolytic lesions with sclerotic margins and an expansile growth pattern involving both maxillae and the mandible. As shown in Figure 1A, the largest lesion was located in the right maxilla and measured approximately 50 × 41 × 47 mm. It occupied nearly the entire right maxillary sinus, causing marked thinning and scalloping of the cortical bone and significant mass effect on the nasal cavity, with obstruction of the right nasal passages and ostiomeatal complex. An impacted tooth was present within the lesion in the region of the right maxillary canine.

**Figure 1:**
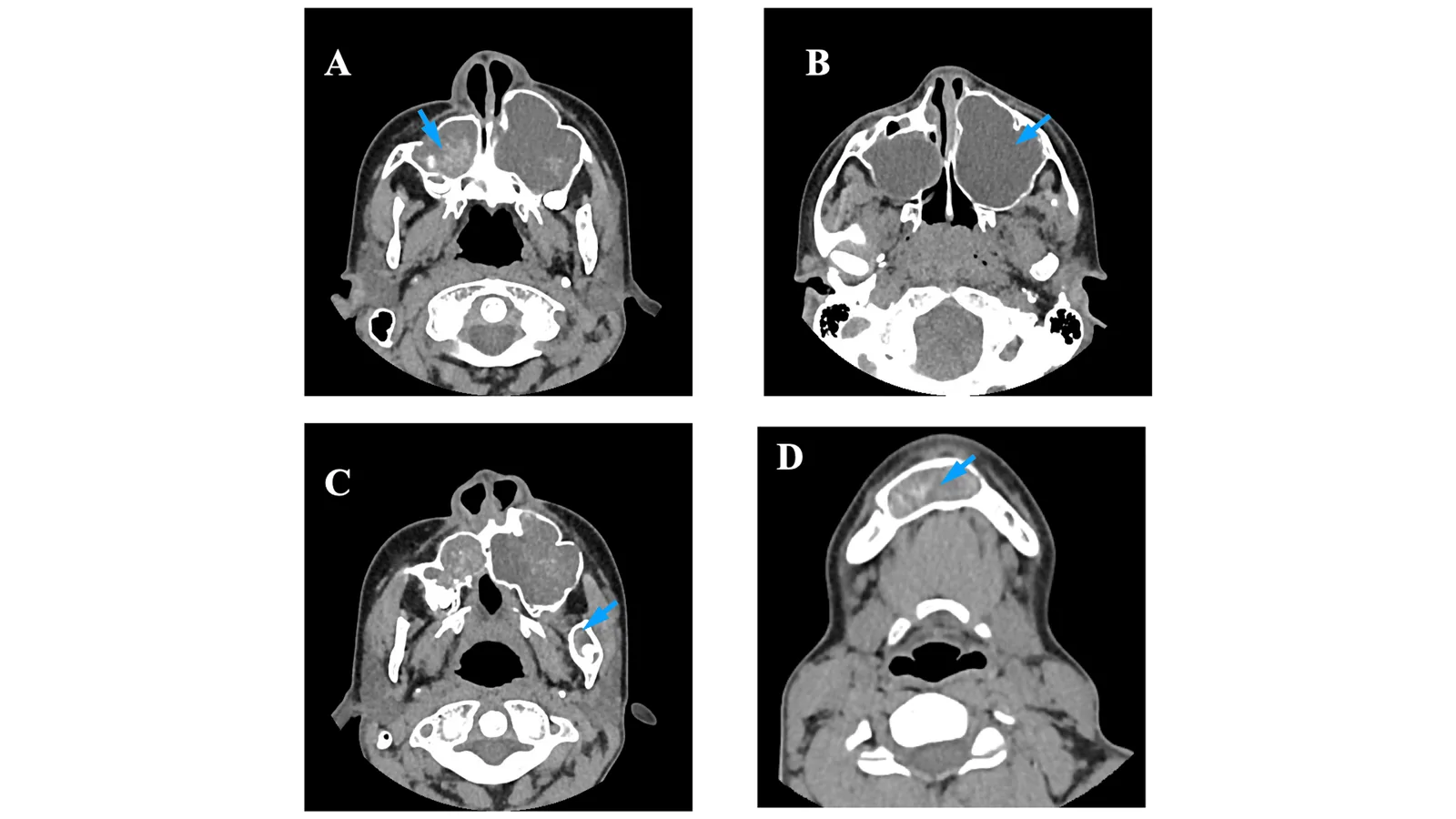
Preoperative computed tomography (CT) of the maxillofacial region demonstrating multiple extensive odontogenic keratocysts. (A) Axial CT image showing an extensive osteolytic lesion involving the right maxilla and occupying nearly the entire right maxillary sinus, with marked thinning and scalloping of the cortical bone. The blue arrowhead indicates the odontogenic keratocyst in the right maxilla. (B) Axial CT image demonstrating an extensive lesion involving the left maxilla and occupying the left maxillary sinus, with marked cortical thinning and elevation of the orbital floor. The blue arrowhead indicates the odontogenic keratocyst in the left maxilla. (C) Axial CT image showing an expansile osteolytic lesion involving the left mandibular angle and associated with an impacted tooth. The blue arrowhead indicates the odontogenic keratocyst in the left mandibular angle. (D) Axial CT image demonstrating an additional expansile lesion involving the anterior mandibular body. The blue arrowhead indicates the odontogenic keratocyst in the anterior mandibular body.

Figure 1B demonstrates a second extensive lesion in the left maxilla, measuring approximately 55 × 44 × 54 mm and occupying the entire left maxillary sinus. It produced pronounced thinning and scalloping of the cortical bone, with possible focal cortical discontinuity, significant mass effect on the nasal cavity, obstruction of the left nasal passages and ostiomeatal complex, and elevation of the left orbital floor. The lesion also involved the impacted left maxillary canine.

As shown in Figure 1C, the third lesion was located in the region of the left mandibular angle and measured approximately 32 × 16 × 41 mm. It caused marked expansion, thinning, and scalloping of the cortical bone and completely encompassed the impacted mandibular third molar.

Figure 1D demonstrates the fourth lesion, which was located centrally and toward the right side of the mandibular body, in the region of the symphysis, and measured approximately 14 × 33 × 28 mm. This lesion also demonstrated marked cortical expansion and thinning and was adjacent to an ectopically positioned mandibular tooth. The multifocal distribution, well-defined margins, expansile character, and association with impacted teeth were highly suggestive of multiple OKCs.

Magnetic resonance imaging (MRI) of the maxillofacial region was subsequently performed with intravenous contrast. Figure 2A demonstrates the bilateral extensive maxillary lesions, while Figures 2B, 2C demonstrate the lesions involving the maxilla on sagittal images. The examination demonstrated four well-defined lesions involving the bilateral maxillae, left mandibular angle, and anterior mandibular body, with marked expansion of the surrounding bone. The lesions showed heterogeneous signal intensity, diffusion restriction, and peripheral enhancement following contrast administration. No infiltration of the surrounding soft tissues was identified. Overall, the MRI findings were consistent with the CT appearance and supported the radiological diagnosis of multiple OKCs.

**Figure 2:**
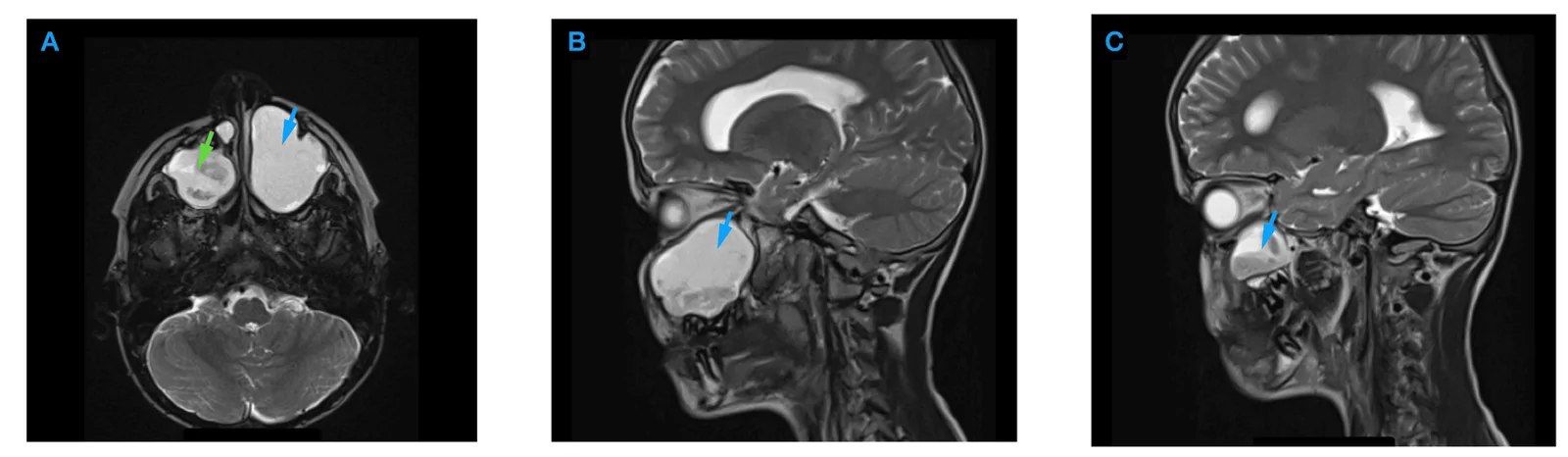
Preoperative magnetic resonance imaging (MRI) of the maxillofacial region demonstrating extensive odontogenic keratocysts. (A) Axial T2-weighted MRI image demonstrating bilateral extensive lesions involving the maxillary sinuses. The blue arrowhead indicates the odontogenic keratocyst in the right maxillary sinus, while the green arrowhead indicates the odontogenic keratocyst in the left maxillary sinus. (B) Sagittal T2-weighted MRI image demonstrating an extensive odontogenic keratocyst involving the maxilla and occupying a large portion of the maxillary sinus. The blue arrowhead indicates the odontogenic keratocyst. (C) Sagittal T2-weighted MRI image demonstrating an extensive odontogenic keratocyst involving the maxilla with extension into the maxillary sinus. The blue arrowhead indicates the odontogenic keratocyst.

The CT examination also demonstrated intracranial calcifications involving the falx cerebri, as shown in Figure 3. Additional skeletal abnormalities were identified on chest radiography, including fusion of the posterior portions of the right fourth and fifth ribs, bifid morphology of the right fifth rib, bifid morphology involving the left fourth and sixth ribs, and possible fusion of the posterior portions of the left sixth and seventh ribs. Left-sided cervicothoracic spinal curvature was also noted.

**Figure 3:**
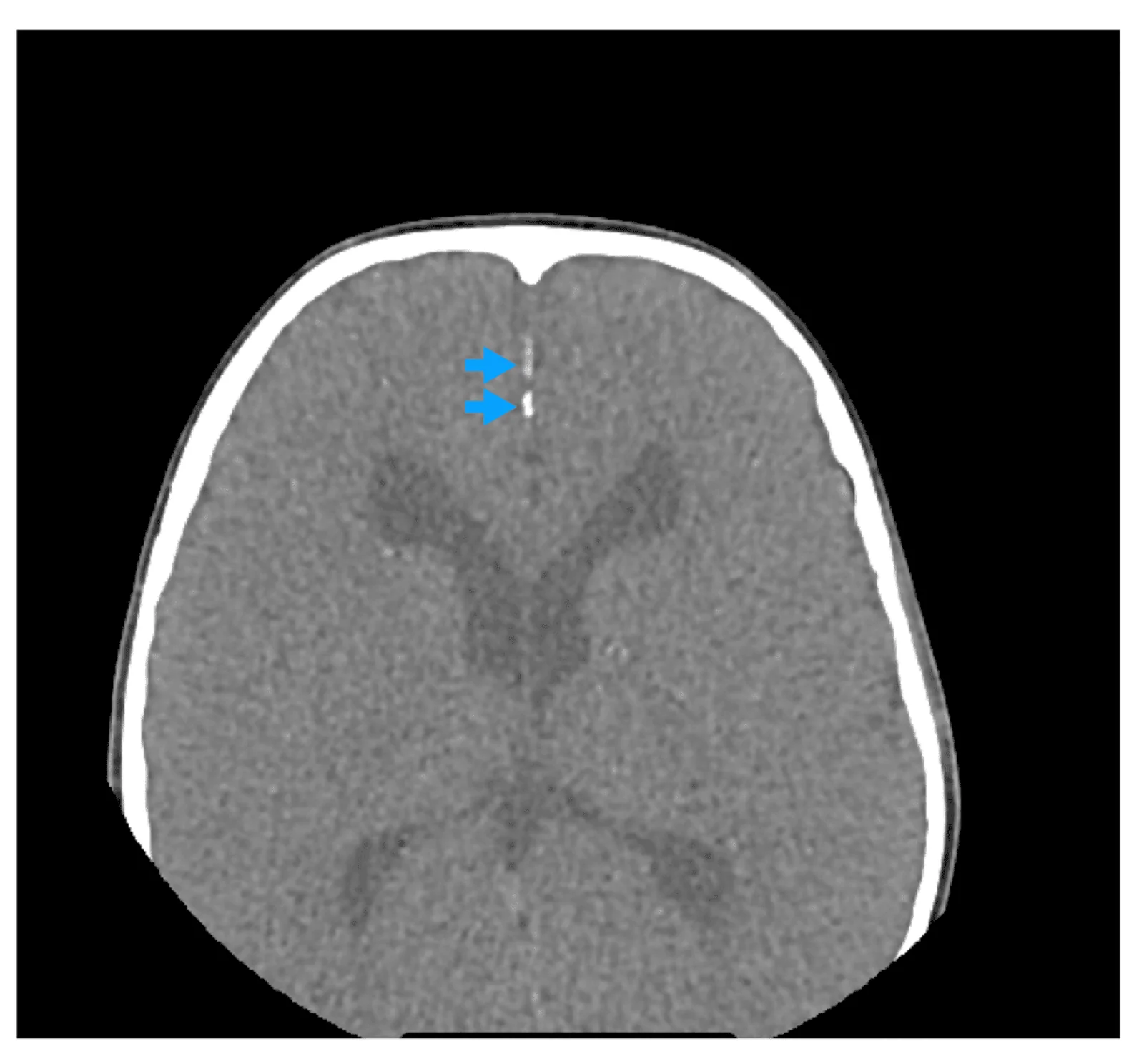
Preoperative computed tomography (CT) of the brain demonstrating calcifications of the falx cerebri. Blue arrows indicate the areas of calcification within the falx cerebri.

Despite the marked radiological mass effect and obstruction of the nasal passages and ostiomeatal complexes, the patient did not report clinically significant nasal obstruction or rhinorrhea, and there was no reported history of sinusitis. Thus, the sinonasal involvement was predominantly a radiological finding rather than a symptomatic ENT presentation.

The combination of multiple extensive OKCs diagnosed before 20 years of age and lamellar calcification of the falx cerebri fulfilled two major diagnostic criteria for GGS according to the clinical management guidelines. The identified rib abnormalities represented an additional minor diagnostic criterion. Based on the presence of two major criteria, a clinical diagnosis of GGS was established [9].

The patient underwent genetic consultation, and both the patient and his family were referred for genetic testing. At the time of the described clinical evaluation, genetic assessment was ongoing and the final results were awaited.

Given the extensive size and multifocal distribution of the lesions, surgical treatment was planned. Although decompression may be considered in pediatric patients with OKCs, the considerable size of the lesions, marked cortical thinning and expansion, involvement of both maxillary sinuses with associated radiological obstruction of the nasal passages and ostiomeatal complexes, and the presence of impacted or ectopically positioned teeth favored definitive enucleation in this case. Because all four lesions could be addressed during the same procedure, complete enucleation allowed simultaneous treatment of the lesions and removal of the associated teeth, while providing tissue for histopathological confirmation. All four lesions were therefore enucleated during a single operative procedure under general anesthesia with nasotracheal intubation.

The corresponding procedure was then performed on the right maxilla. The lesion was completely enucleated together with the impacted maxillary canine, while the sinus mucosa was preserved. The posterior impacted tooth and associated enlarged follicular tissue were also removed. The operative fields were irrigated and hemostasis was achieved before repositioning and suturing the mucoperiosteal flaps.

The mandibular lesions were treated during the same procedure. The lesion in the left mandibular angle was exposed through an intraoral approach and completely enucleated together with the associated impacted tooth. The anterior mandibular lesion was accessed through a gingival incision, followed by removal of the cystic lesion together with the associated ectopically positioned tooth. All excised lesions and associated dental tissues were submitted for histopathological examination.

Histopathological examination confirmed the diagnosis of OKCs in all excised lesions. The postoperative course was uneventful, with no reported complications. The patient was referred for continued multidisciplinary follow-up following the clinical diagnosis of GGS, with particular attention to the risk of recurrence and the development of additional lesions.

## Discussion

This case highlights the importance of multiple OKCs as a potential early manifestation of GGS in children. In our 13-year-old patient, four extensive OKCs involving both maxillae and the mandible represented the predominant clinical manifestation of the syndrome, despite the absence of characteristic cutaneous abnormalities. Selected clinical and therapeutic features of previously reported pediatric cases of GGS-associated multiple OKCs are summarized in Table 1 [10,11]. Multiple OKCs, particularly in children and young adults, are an important clinical clue to the diagnosis of GGS and should prompt evaluation for additional syndromic manifestations [5,8]. The relatively limited clinical symptoms in our patient, despite the considerable extent of the lesions, further emphasize that clinically silent or minimally symptomatic OKCs may progress substantially before diagnosis.

**Table 1:** Comparison of selected clinical and therapeutic features of pediatric patients with Gorlin-Goltz syndrome and multiple odontogenic keratocysts. OKCs: Odontogenic keratocysts; GGS: Gorlin-Goltz syndrome

Feature	Santander et al. [10]	Boos Lima et al. [11]	Present case
Age at diagnosis	8 years	9 years	13 years
Number/distribution of OKCs	Multiple; mandible and maxilla	Multiple; mandible and maxilla	Four; bilateral maxilla and mandible
Associated dental findings	Lesions associated with tooth crowns	Ectopic/displaced teeth	Multiple impacted/ectopic teeth
Maxillary sinus involvement	Not reported as a major feature	Not reported as a major feature	Bilateral near-total involvement
Treatment	Surgical removal of cysts and affected teeth	Marsupialization and enucleation	Single-stage enucleation of all four lesions with removal of associated teeth
Genetic findings	Germline PTCH1 variant	Not reported	Genetic testing ongoing
Follow-up	5 years	Follow-up reported	Long-term follow-up unavailable at our institution
Distinctive feature	Recurrent/multifocal disease during dental maturation	Multiple OKCs with numerous GGS manifestations	Extensive bilateral maxillary sinus involvement despite limited symptoms

The age and extent of the lesions in our patient are consistent with the characteristic early presentation of jaw cysts in GGS. In a series of 105 individuals with nevoid basal cell carcinoma syndrome, Kimonis et al. described jaw keratocysts as a common manifestation of the syndrome, together with calcification of the falx cerebri and skeletal abnormalities involving the spine and ribs [12]. The authors also demonstrated considerable variability in the clinical expression of the syndrome among affected individuals [12]. In comparison, our patient presented at 13 years of age with four extensive OKCs involving both maxillae and the mandible. The lesions demonstrated marked cortical expansion, displacement of adjacent anatomical structures, and association with impacted teeth. This extensive multicentric involvement at a young age illustrates the potential severity of syndromic OKCs and supports the importance of early radiological assessment in children presenting with multiple jaw lesions or unexplained facial asymmetry.

The diagnosis of GGS in our patient was established according to the currently accepted clinical diagnostic criteria. The clinical management guidelines state that a diagnosis can be established by the presence of two major criteria, one major criterion with molecular confirmation, or one major criterion together with two minor criteria [9]. Our patient fulfilled two major criteria: OKCs diagnosed before the age of 20 years and lamellar calcification of the falx cerebri. In addition, the identified rib abnormalities represented a minor diagnostic criterion [9]. The left-sided cervicothoracic spinal curvature constituted an additional skeletal radiological abnormality, although it was not required to establish the diagnosis. Importantly, the absence of characteristic cutaneous manifestations did not preclude the diagnosis, as the clinical expression of GGS is variable and individual manifestations may become apparent at different ages [9,12]. Thus, the combination of multiple early-onset OKCs and intracranial calcification provided sufficient evidence for a clinical diagnosis of GGS, while genetic testing was pursued for further molecular characterization.

The treatment of multiple extensive OKCs in patients with GGS remains challenging, particularly when lesions involve several anatomical sites and are associated with substantial expansion of the surrounding bone. Treatment strategies include conservative approaches such as decompression, as well as enucleation with or without adjunctive procedures. In a retrospective study of 102 keratocysts, including 32 syndromic lesions associated with nevoid basal cell carcinoma syndrome, Noy et al. reported a significantly higher recurrence rate in syndromic than in sporadic lesions (47% vs. 20%), with recurrence diagnosed approximately three years after treatment on average [13]. In a separate retrospective analysis of 145 histopathologically confirmed keratocystic odontogenic tumors, Titinchi and Nortjé also evaluated recurrence in relation to clinical and radiographic parameters, including the presence of NBCCS [14]. Conversely, Ribeiro-Júnior et al., in a cohort of 40 OKCs including 13 syndromic lesions, found no significant difference in recurrence-free periods between syndromic and nonsyndromic lesions or among the treatment groups evaluated [15]. These findings indicate that the recurrence behavior of OKCs in GGS is complex and that treatment outcomes cannot be predicted solely on the basis of syndromic status. In our patient, all four lesions were treated by complete enucleation during a single surgical procedure. The decision to perform simultaneous treatment was influenced by the multifocal nature of the disease and the considerable size and anatomical extent of the lesions. The bilateral maxillary lesions required management of the maxillary sinuses and associated impacted teeth, while the mandibular lesions were treated during the same anesthetic procedure. Given the documented risk of recurrence and the possibility of developing additional OKCs, long-term clinical and radiological surveillance is essential. Noy et al. reported that recurrence was most commonly detected approximately three years after treatment, supporting the need for prolonged follow-up [13].

The present case also illustrates the diagnostic value of comprehensive imaging in children with multiple OKCs. Initial imaging demonstrated the full extent and multicentric distribution of the lesions, while subsequent MRI further characterized their relationship to adjacent structures and excluded significant soft-tissue infiltration. Additional imaging of the skull and chest identified intracranial calcification and rib abnormalities that contributed directly to fulfilling the diagnostic criteria for GGS. This emphasizes that the recognition of multiple OKCs should not be regarded solely as a local maxillofacial problem but rather as a potential marker of an underlying multisystem disorder. Early recognition of GGS is clinically important because establishing the diagnosis allows appropriate surveillance, genetic counselling, and timely evaluation of additional manifestations of the syndrome [9]. In children presenting with multiple OKCs, particularly in the absence of obvious cutaneous findings, a systematic assessment for other diagnostic criteria may therefore substantially shorten the diagnostic pathway.

A limitation of this report is the lack of long-term follow-up data regarding recurrence and functional outcomes, as the patient continued follow-up at his local place of residence after the postoperative period at our referral center.

## Conclusions

Multiple OKCs may be the first clinically apparent manifestation of GGS in children. Their presence, particularly at a young age and in a multicentric distribution, should prompt comprehensive evaluation for additional skeletal and intracranial abnormalities, even when characteristic skin manifestations are absent. Early recognition of the syndrome enables appropriate multidisciplinary management and long-term surveillance. In patients with extensive jaw involvement, individualized surgical planning is essential to address the lesions while preserving anatomical structures and minimizing functional morbidity.
